# Supplementary figures and images for: Mortality and associated risk factors in patients with blood culture positive sepsis and acute kidney injury requiring continuous renal replacement therapy—A retrospective study
Source: PLoS One. 2021 Apr 5;16(4):e0249561. doi: 10.1371/journal.pone.0249561 (PMC8021149; doi:10.1371/journal.pone.0249561)

## Slide 1
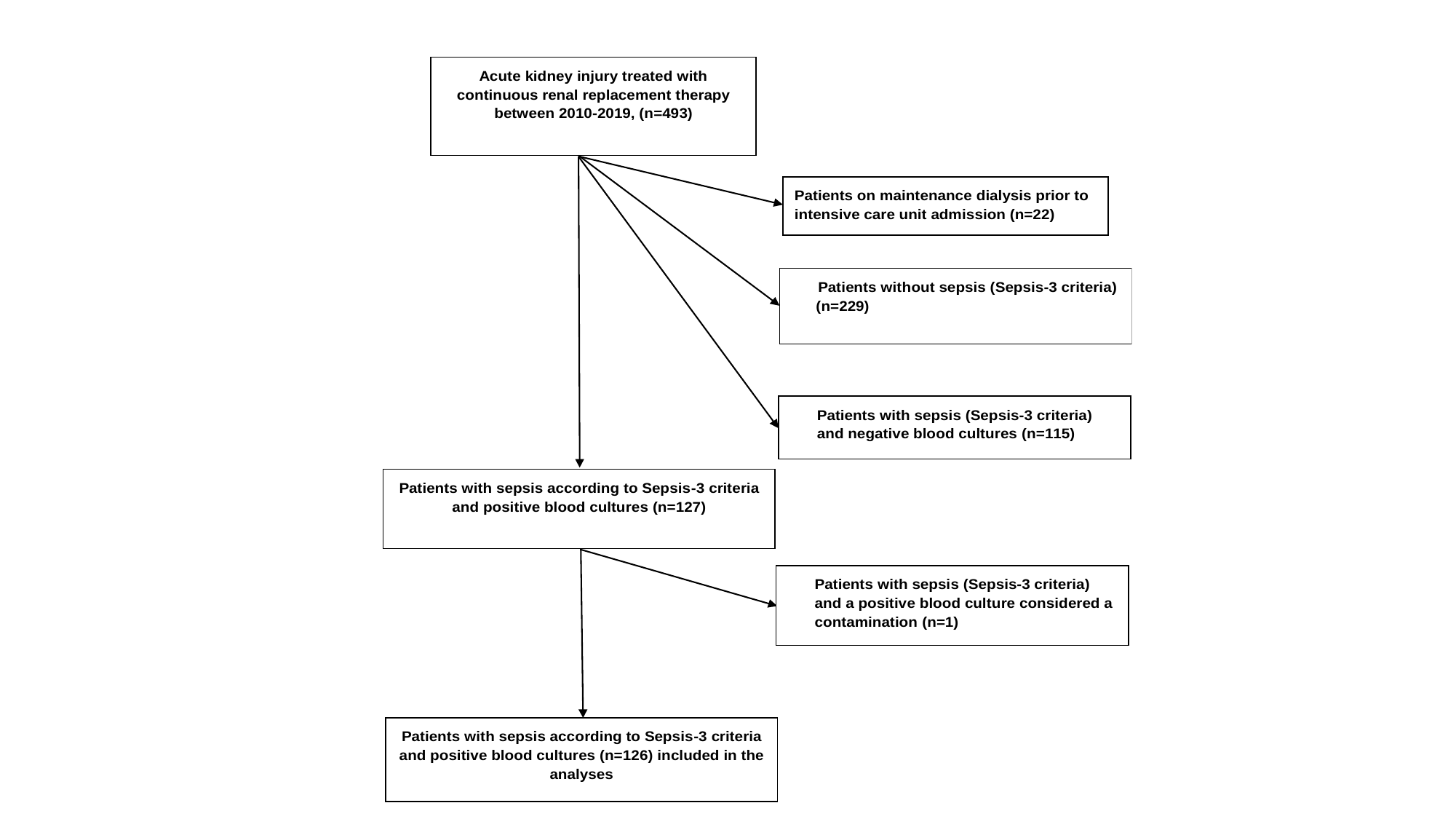

Supplement: S1 Fig — (PPTX) [file pone.0249561.s001.pptx]
