# Supplementary material for: Mortality and associated risk factors in patients with blood culture positive sepsis and acute kidney injury requiring continuous renal replacement therapy—A retrospective study
Source: PLoS One. 2021 Apr 5;16(4):e0249561. doi: 10.1371/journal.pone.0249561 (PMC8021149; doi:10.1371/journal.pone.0249561)
Supplement: S1 Table — (DOCX) [file pone.0249561.s002.docx]

Supplemental Table 1. Antimicrobial regimens used: 1^st^ empiric treatment; specific treatment; and all antimicrobial regimes used during intensive care unit (ICU) stay.

| **Antimicrobial regimen** | **Empiric treatment** | **Specific treatment** | **Entire ICU stay** |
| --- | --- | --- | --- |
| Cefuroxime | 21 (17%) | 3 (2%) | 21 (17%) |
| Ceftriaxone | 31 (25%) | 16 (13%) | 43 (34%) |
| Ceftazidime | 1 (1%) | 1 (1%) | 2 (2%) |
| Penicillin G | 3 (2%) | 13 (10%) | 14 (11%) |
| Ampicillin | 1 (1%) | 0 (0%) | 2 (2%) |
| Cloxacillin | 3 (2%) | 25 (20%) | 26 (21%) |
| Piperacillin/Tazobactam | 46 (37%) | 29 (23%) | 48 (38%) |
| Meropenem | 27 (21%) | 41 (33%) | 63 (50%) |
| Imipenem | 1 (1%) | 1 (1%) | 3 (2%) |
| Clindamycin | 3 (2%) | 30 (24%) | 40 (32%) |
| Ciprofloxacin | 1 (1%) | 4 (3%) | 13 (10%) |
| Levofloxacin | 6 (5%) | 5 (4%) | 12 (10%) |
| Moxifloxacin | 7 (6%) | 11 (9%) | 19 (15%) |
| Vancomycin | 6 (5%) | 5 (4%) | 37 (29%) |
| Gentamycine | 1 (1%) | 5 (4%) | 6 (5%) |
| Tobramycin | 0 (0%) | 1 (1%) | 1 (1%) |
| Amikacin | 2 (1%) | 1 (1%) | 2 (2%) |
| Rifampicine | 1 (1%) | 20 (16%) | 20 (16%) |
| Teicoplanin | 0 (0%) | 1 (1%) | 1 (1%) |
| Linezolide | 0 (0%) | 1 (1%) | 5 (4%) |
| Metronidazole | 4 (3%) | 3 (2%) | 5 (4%) |
| Tigecyclin | 1 (1%) | 0 (0%) | 7 (6%) |
| Fluconazole | 3 (2%) | 6 (5%) | 32 (25%) |
| Anidulafungin | 1 (1%) | 2 (2%) | 17 (13%) |
| Micafungin | 0 (0%) | 2 (2%) | 8 (6%) |
| Caspofungin | 0 (0%) | 0 (0%) | 3 (2%) |
