# Supplementary material for: Mortality and associated risk factors in patients with blood culture positive sepsis and acute kidney injury requiring continuous renal replacement therapy—A retrospective study
Source: PLoS One. 2021 Apr 5;16(4):e0249561. doi: 10.1371/journal.pone.0249561 (PMC8021149; doi:10.1371/journal.pone.0249561)
Supplement: S2 Table — For comparability, the hazard ratios are for standardized variables with mean 0 and SD 1. (DOCX) [file pone.0249561.s003.docx]

Supplemental Table 2. Factors associated with mortality in univariate models. For comparability, the hazard ratios are for standardized variables with mean 0 and SD 1.

| Variable | HR (95% CI) | P-value |
| --- | --- | --- |
| Age | 1.30 (1.02-1.65) | 0.03 |
| History of heart failure | 2.01 (1.25-3.23) | 0.004 |
| Immunosupression | 2.84 (1.68-4.80) | 0.0001 |
| Peak SOFA score | 1.34 (1.05-1.71) | 0.02 |
| APACHE II score | 1.64 (1.29-2.09) | <0.0001 |
| SAPS II score | 1.70 (1.33-2.17) | <0.0001 |
| C-reactive protein at admission | 0.65 (0.51-0.83) | 0.0004 |
| INR at admission | 1.39 (1.17-1.66) | 0.0002 |
| Lactate at admission | 1.45 (1.20-1.76) | 0.0001 |
| pH at admission | 0.67 (0.54-0.82) | 0.0002 |
| Bicarbonate at admission | 0.76 (0.59-0.96) | 0.02 |
| Lactate at CRRT initiation | 1.55 (1.33-1.81) | <0.0001 |
| pH at CRRT initiation | 0.63 (0.51-0.77) | <0.0001 |
| Bicarbonate at CRRT initiation | 0.61 (0.48-0.78) | 0.0001 |
| Base excess at CRRT initiation | 0.62 (0.49-0.78) | <0.0001 |

SD=standard deviation; HR=hazard ratio; CI=confidence interval; SOFA=sequential organ failure assessment; APACHE-II=acute physiology and chronic health evaluation II; SAPS-II=simplified acute physiology II; INR=international normalized ratio; CRRT=continuous renal replacement therapy
